# Acquired Hemophilia in an Elderly Patient with Non-Small Cell Lung Cancer: a Case Report

**DOI:** 10.1007/s42399-022-01330-x

**Published:** 2022-11-23

**Authors:** Antonio Dulcetti, C. Bruscia, D. M. Malena, R. Benvenuto, A. Martocchia, A. Sentimentale, L. Tafaro, M. Rocchietti March, P. Martelletti

**Affiliations:** UOC Medicina d’Urgenza, Azienda Ospedaliera-Universitaria Sant’Andrea, Università Degli Studi Di Roma La Sapienza, Via di Grottarossa, 1035, Rome, 00189 Italy

**Keywords:** Acquired hemophilia A, Recombinant activated factor VII, Case report, Bleeding, Paraneoplastic

## Abstract

Acquired hemophilia A (AHA) is a rare autoimmune disease caused by autoantibodies against coagulation factor VIII and characterized by spontaneous hemorrhage in patients with no previous family or personal history of bleeding. We report here a case of AHA that occurred in the Department of Medicina D’Urgenza in Sant’Andrea Hospital in a patient with previous diagnosis of NSLC. The aim of this article is to allow a more comprehensive knowledge of AHA that both for the rarity and the poor literature is underdiagnosed; for all these reasons, it is important that different specialists, like emergency specialists, experts in internal medicine, hematologists, and oncologists, acquire a more complete knowledge of the clinical and laboratory features of this disease, allowing an early diagnosis crucial for the evolution of the coagulopathy.

## Introduction

Hemophilia A is defined as a factor VIII deficiency with a X-linked transmission: genes are located on the X chromosome so hemophilia almost exclusively affects the male population, with outset in the pediatric age. The diagnosis can be found between 36 months, in mild-moderate cases, and 1 month in severe cases with factor VIII activity below 1% [1].

The beginning of the clinical manifestations is substantially hemorrhagic such as hemarthrosis, retroperitoneal bleeding, or muscle hematomas.

AHA (acquired hemophilia A) is a rare adulthood disorder with a similar clinical picture, with an incidence of 1 per 1 million per year, which essentially affects elderly population [2].

Although the pathophysiological mechanism underlying this alteration is still being defined, the production of circulating autoantibodies inhibiting endogenous factor VIII seems to be involved [3, 4].

In about half of the cases, AHA is idiopathic, but in a significant proportion it can be associated with neoplasia (11.8%), autoimmune diseases (13.4%), infection (3.8%), and pregnancy (8.4%) [5].

A case has also been reported to be associated with SARS-CoV 2 infection [6].

The main clinical manifestation of AHA is hemorrhage, present in more than 90% of patients at onset; the bleeding is spontaneous and severe in 70% of cases. The most common bleeding sites involved are subcutaneous tissue, followed by gastrointestinal hemorrhages and muscular hematomas, and less frequently genitourinary, retroperitoneal, and even intracranial.

Hemarthrosis, which are typical manifestations of congenital hemophilia A, are rare in the acquired forms. Only in a minority of cases (only about 7%) AHA has not been associated with bleeding and is diagnosed while investigating for other pathologies or in view of an invasive procedure [5, 7].

AHA is considered a serious disease not only due to the incidence of severe bleeding but also due to the high mortality rate, estimated at over 10% in patients over 65 years [8].

According to the “International recommendations on the diagnosis and treatment of acquired hemophilia A,” in order to make the diagnosis it is necessary to highlight an isolated lengthening of the activated partial thromboplastin time (aPT) and normal prothrombin time (PT) and an isolated deficit of factor VIII; additional tests which are used in diagnostics include timed plasma mixing test, lupus anti-coagulant (LAC), and Bethesda assay for inhibitor antibody quantification [4].

Treatment includes immunosuppressive therapy and the use of recombinant activated factor VII or recombinant porcine factor VIII as a hemostatic drug [3, 9].

## Case Description

We report the case of an 84-year-old patient with AHA who presented replenished bleeding in the muscle and liver tissue.

The patient was admitted in the emergency room of “St. Andrea Hospital” for acrocyanosis and pain at the right upper limb. He also reported dyspnea the week prior to emergency room access and denied any traumatic event to the affected limb.

He presented a pathological history of chronic obstructive pulmonary disease (COPD) and a pulmonary adenocarcinoma in the right lower lobe, which was already been treated with chemotherapy, and in a state of follow-up by another hospital, hypertension and previous acute coronary syndrome in 2018.

The home therapy included ramipril, cardioaspirin, bisoprolol, spironolactone, pantoprazole, and furosemide.

The patient reported having stopped clopidogrel about 2 weeks before admission under the advice of his doctor, due to the appearance of a large hematoma on the affected limb.

### Physical examination at the entrance

“Alert patient, oriented, pale skin, not sweaty, moderate hemodynamic compensation, right upper limb increased in volume, edematous, brachial and radial pulse not very detectable but present.”

Vital signs were BP 100/60 mmHg, HR 90 bpm, and SvO2 99%.

Hematochemical tests documented a normocytic normochromic anemia, HB 7.2 g/dL, RBC 2.500.000/μL, PLT 263,000/μL, and WBC 7.090/μL with appropriate formula, liver and kidney functions in normal range, and PT 12.10 s INR 1.08, aPTT 2.24 (70 s), and D-dimer 2856 ng/mL.

**Fig. 1:**
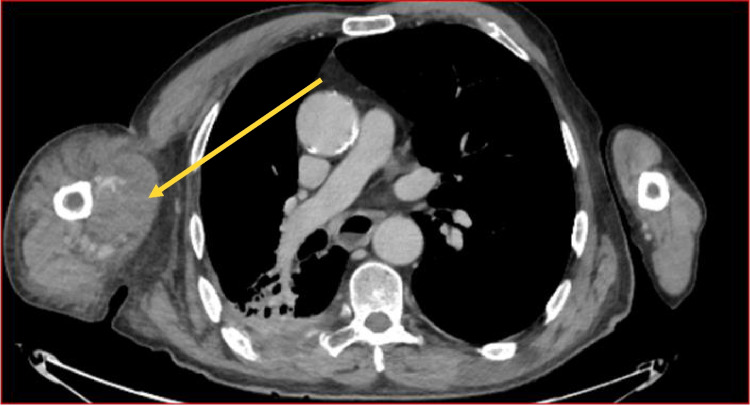
CT showing a hematoma of the right upper limb

During the period in the emergency room, a transfusion with 1U of concentrated red blood cells was administered and a chest and upper limb angio-CT (Fig. 1) was requested which documented:

“… Is reported the presence of a volumetric increase in the right arm, compared to the contralateral one, due to the presence of a blood collection in the context of the biceps muscle in the perihumeral site…After contrast medium is observed a blood spreading of contrast medium as for bleeding in the active phase. Collaterally to the VII hepatic segment area of focal hypodensity of 3.5 cm in which context blood spreading is observed as for bleeding in the active phase.”

An upper limb arteriography was therefore performed and a superselective catheterization, with subsequent embolization.

Despite the procedure, the patient continued to have variability in his hemoglobin and coagulation values.

After that, the patient was admitted to the Emergency Medicine Department, and blood chemistry tests were repeated, confirming the presence of hemoglobin values of 7.1 g/dL despite transfusion support, normal liver and kidney function, normal inflammation indices, PT 12.08 s (INR 1.07), and aPTT 2.51 (VN 0.8–1.2)/79 s.

From an interview with the patient and with his son, we learned that in the preceding months the patient had made 4 admissions to different hospitals for episodes of acute anemia, without mention of any trauma.

It was decided to carry out a new transfusion and a thorough coagulation screen was performed (Table 1), showing an isolated factor VIII deficiency (0.7%, reference range 50–150), LAC negative, and mixing test was not performed.

**Table 1:** Coagulation parameters of the patient

PT	1.16
Normal range	79%
0.9–1.2	13.9 s
70–120%	
10–13 s	
aPTT	**74s**
n.r	**2.36 ratio**
25–35 s	
08–1.2	
D-Dimer	**5498 ng/mL**
nr < 243	
Fibrinogen	446 mg/mL
n.r 200–400	
Factor VIII	**0.7%**
Nr 50–150%	
Factor IX	89%
65–150%	

At the time of diagnosis, the patient decided to resign against medical advice, relying on home palliative care.

## Discussion

AHA represents a very rare and potentially lethal disease which, unlike hemophilia A, usually arises in adulthood.

This pathology requires a multidisciplinary approach that involves experts in internal medicine, hematologists, urgency specialists, immunologists, radiologists, and laboratory doctors.

The importance of diagnosis, and in particular of early diagnosis, is underlined by the high mortality rate of patients suffering from this condition, as the probability of severe bleeding concerns 70% of these patients.

The diagnosis, as mentioned, starts from the suspicion of prolonged aPTT in the absence of a known coagulopathy, therapy with heparin e.v., and LAC negative, confirmed by the dosage of coagulation factors, with evidence of FVIII deficiency.

Therapy, as mentioned, can be aimed at turning off the immune stimulus that leads to the production of anti-FVIII antibodies, such as corticosteroids associated with cyclophosphamide, or according to more recent studies with monoclonal autoantibodies such as rituximab (anti CD20).

Another interesting therapeutic approach, which we can define as hemostatic and that concerns approximately 70% of patients with AHA, involves the use of FVIIa or activated prothrombin complex concentrate or recombinant porcine factor VIII. [3, 4]; however, it is advisable to contact centers specialized in the treatment of hemophilia.

The diagnostic challenge consists in the multidisciplinary approach, and in the polypharmacy and polypathology typical of the elderly population that can mask the clinical picture.

The aim of this article is to allow a more comprehensive knowledge of AHA that both for the rarity and the poor literature is underdiagnosed; for all these reasons, it is important that different specialists, like emergency specialist, specialists in internal medicine, hematologists, and oncologists, acquire a more complete knowledge of the clinical and laboratory features of this disease, allowing an early diagnosis crucial for the evolution of the coagulopathy.

In conclusion, AHA in a significant proportion can be associated with malignancy, autoimmune diseases, and infections; the rapidity of the diagnosis could lead to a rapid resolution of the coagulopathy and also to immediate treatment of the underlying causes.

## Data Availability

The datasets generated and/or analyzed during the current study are available from the corresponding author on reasonable request.

## Code Availability

NA.
